# Method for efficient calculating earth pressure of retaining wall considering plant transpiration

**DOI:** 10.1038/s41598-023-42411-8

**Published:** 2023-09-16

**Authors:** Junhui Zhang, Huiren Hu, Wei Fu, Junhui Peng, Feng Li, Le Ding

**Affiliations:** 1 National Engineering Research Center of Highway Maintenance Technology, Changsha University of Science & Technology, Changsha, 410114 Hunan China; 2 School of Traffic & Transportation Engineering, Changsha University of Science & Technology, Changsha, 410114 Hunan China; 3 CCCC Second Highway Consultants Co., Ltd., Wuhan, 430052 Hubei China; 4 School of Civil Engineering and Architecture, East China Jiaotong University, Nanchang, 330013 Jiangxi China; 5 Hunan Engineering Research Center of Structural Safety and Disaster Prevention for Urban Underground Infrastructure, Hunan City University, Yiyang, 413000 Hunan China

**Keywords:** Environmental sciences, Civil engineering

## Abstract

An accurate estimation of earth pressure on retaining walls is imperative to achieving its design. This paper presents an analytical method framework that considers the effect of plant transpiration relative to the traditional calculation approaches. Specifically, a closed-form solution for one-dimensional steady unsaturated flow considering plant transpiration is incorporated into a representation of effective stress to obtain the changes in matric suction, and effective stress. The representations are used to extend Hooke’s law and Rankine’s earth pressure theory to determine at-rest, active, and passive earth pressures. Subsequently, the analytical method is used in a series of analysis case studies on the influence of root architecture types, transpiration rates, and soil types on earth pressure, to reveal that it can rapidly obtain the earth pressure. Notably, the effect of plant transpiration on earth pressure is significant. Furthermore, it is found that soil types and transpiration rates have a larger influence than root architecture types. Collectively, the research not only reveals the effect of plant on earth pressure for retaining wall, but also provides a theoretical basis for further exploration of the contribution of plants to the stability of retaining wall.

## Introduction

As a widely used support structure, the retaining wall plays a vital role in geotechnical engineering, such as slope support engineering, foundation pit supporting engineering, etc. The earth pressure it bears is the key index to evaluate its stability and always arouses great attention^1,2^. It, therefore, becomes important to determine the earth pressure exactly. The most famous methods for calculating earth pressures are Coulomb’s and Rankine’s earth pressure theories seen as limit equilibrium methods. The limit analysis method^3–5^ and the slip method^2,6–8^ were also proposed successively. These researches provide alternative methods to calculating the earth pressures of the backfill behind retaining wall and promoting the researcher’s understanding of the earth pressure loading mechanism.

As the earth pressure sources to retaining wall, the soil is composed of a three-phase medium, i.e., solid, gas, and liquid. Because of the complexity of the three-phase medium, it is often treated as a two-phase medium of solid and liquid, i.e., saturated soil, and, in the field of geotechnical engineering, a large number of research theories are carried out for saturated soil, such as the steady calculation of slope^9^, foundation^10^, retaining wall^11,12^, etc. However, when the soil is unsaturated, it has significantly different properties from the saturation one^13,14^. And the contact surface between the three phases of the soil will produce a force that changes the strength of the soil, referred to as matric suction. Many researchers have studied how matric suction changes soil strength. Fredlund et al.^13^ and Bishop^15^ gave the different shear strength theory of unsaturated soil by combining with a large number of experimental data and effective stress principle respectively. Based on unsaturated shear strength theory, traditional methods for calculating earth pressure have been extended^16–20^. Meanwhile, the conditions, i.e., rainfall^20–22^, groundwater^5,23^, and evaporation^4,5^, that can change the unsaturated state of the soil are often considered in these theories, and the results show that the unsaturated state has a significant effect on the earth pressure of retaining wall.

**Figure 1:**
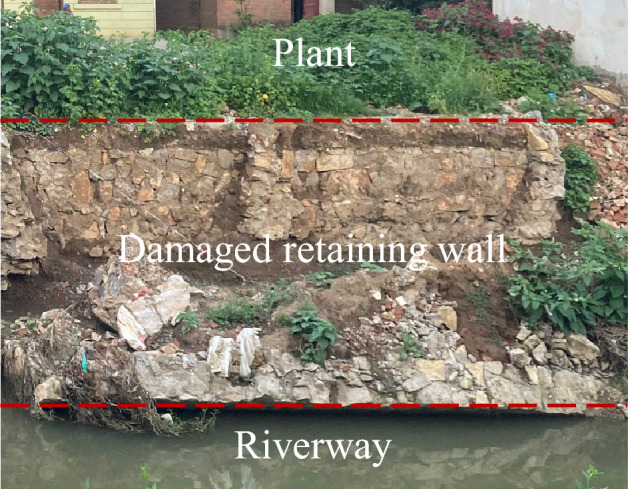
Plant growth of backfill behind retaining wall.

However, the influence of plant growing behind the retaining wall (Fig. 1) on earth pressure is ignored in the existing theoretical methods^1–8^. In recent years, plant has been shown to significantly change the state of unsaturated soil^24,25^. Feng et al.^26^ conducted dimensional analysis to explore dimensionless numbers controlling pore water pressure (PWP) distributions in vegetated slope. Liu et al.^27^ performed a parametric study to investigate hydraulic effect of vegetation on shallow slope stability with different root architectures, and calculated results show the exponential root architecture has higher ability to maintain shallow slope stability than the parabolic one. Zhu et al.^28^ evaluates the effects of root morphology on the enhancement of induced suctions in soil grounds due to transpiration and the results show that the exponential and triangular root networks enhance soil suctions more remarkably than the uniform and parabolic patterns. In a word, plant has a significant effect on matric suction, and the main factors are root structure and transpiration rate. Therefore, it is necessary to expand the existing calculation theory of earth pressure so that it can consider the contribution of plant transpiration.

Several numerical, analytical, and experimental studies have been performed to investigate the effect of plant transpiration on matric suction. Leung and Ng^29^ experimented on a vegetated slope to analyze the effect. Nyambayo and Potts^30^ presents a root water uptake model which has been coded into a finite element program that can perform coupled (mechanical and fluid flow behaviour) analyses. Zhu and Zhang^31^ used random field theory to model the maximum transpiration rate varies spatially and then the effect was studied by finite element software (FlexPDE). Ng et al.^32^ developed analytical solutions for calculating matric suction in an infinite unsaturated slope with different root architectures. Liu et al.^33^ derived a new analytical solutions of PWP distributions in a vegetated multi-layered slope considering the effects of roots on water permeability. It is found that the model experiments need to consume a lot of time and money, e.g., test equipment, model making, data monitoring, etc. The numerical simulation is very complex and then a high level of expertise is required. As a less complex yet reliable alternative, an analytical method for the matric suction is chosen in this paper.

This paper presents an analytical framework to determine active, at-rest, and passive earth pressures of unsaturated soils considering plant transpiration. A closed-form solution for one-dimensional steady unsaturated flow considering plant transpiration was proposed by Ng et al.^32^, which is adapted to the calculation of matric suction for the backfill soil. This solution is incorporated into a representation of effective stress to obtain shear strength. Afterwards, the representation is used to extend Hooke's law and Rankine's earth pressure theory, leading to the determination of at-rest, active, and passive earth pressure for unsaturated soils considering transpiration. Finally, the proposed analytical method is used in a series of cases to study the influence of plant transpiration on earth pressure. This work is the first attempt to develop an analytical method for unsaturated earth pressure considering plant transpiration. The proposed method provides a useful and robust support for assessing the effectiveness of the plants on the stability of the unsaturated earth retaining wall.

### The effective stress and shear strength models of unsaturated soil

In this paper, based upon Bishop’s effective stress expression^15^, the effective stress for unsaturated soils can be defined as Eq. (1).

1 $$\sigma^{\prime} = \left( {\sigma - u_{a} } \right) + \chi \left( {u_{a} - u_{w} } \right)$$

where the first term on the right of the equation is the net stress acting on the soil. The second term is the stress contribution due to matric suction (CMS). σ and σ′ are the total and effective stress, respectively, and *u*_*a*_ represents the pore-air pressure, which is assumed to be equal to the atmospheric pressure and set to zero in this study (*u*_*a*_ = 0)^1^. χ is an effective stress parameter, which is related to the saturation degree of soil. Vanapalli and Fredlund^34^ defined χ when the matric suction range is 0–1500 kPa as:

2 $$\chi = \left( {\frac{{\theta - \theta_{r} }}{{\theta_{s} - \theta_{r} }}} \right)$$

where* θ* is soil water content. *θ*_*r*_ and *θ*_*s*_ are residual water content and saturated water content, respectively.

By applying the effective stress Eq. (1) to the classical Mohr–Coulomb failure criterion, the shear strength failure envelope of unsaturated soil can be expressed in Eq. (3)^1^.

3 $$\tau_{f} { = }c^{\prime} + \left[ {\left( {\sigma - u_{a} } \right)_{f} + \chi \left( {u_{a} - u_{w} } \right)} \right]\tan \varphi^{\prime}$$

where *C'* is effective cohesion. $\varphi^{\prime}$ is the effective angle of internal friction.

**Figure 2:**
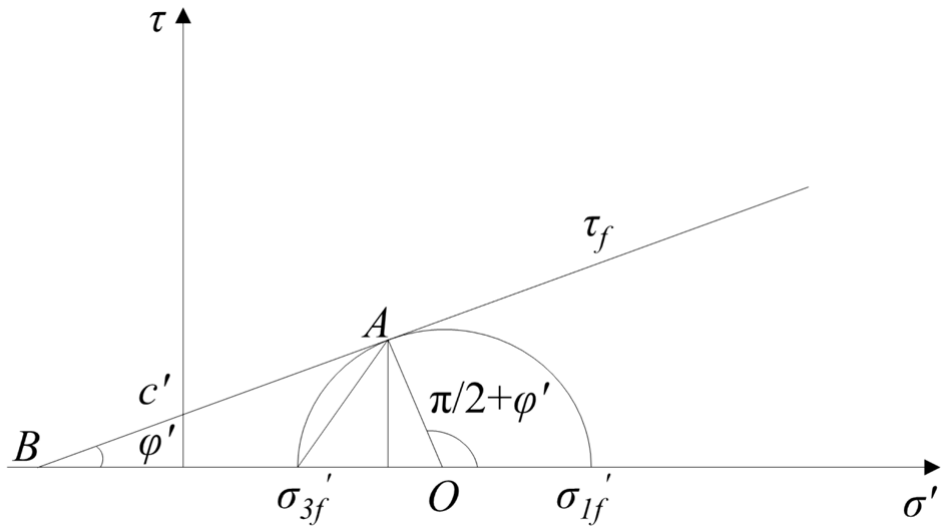
Schematic diagram of failure criterion of unsaturated soil^1^.

The criterion is shown in Fig. 2. The state of soil is explained by the position relationship between the stress circle and the failure envelope. The critical state is that the stress circle is tangent to the failure envelope. When the stress circle is within the failure envelop, the soil will not be damaged. When the stress circle exceeds the scope of the failure envelope, the soil mass has already been damaged. By the geometric relationship in Fig. 2, the relationship between the maximum principal stress $\sigma_{1f}{\prime}$ and the minimum principal stress $\sigma_{3f}{\prime}$ can be solved to assess the state of the soil.

4a $$\sigma_{1f}{\prime} { = }\sigma_{3f}{\prime} {\text{tan}}^{2} \left( {\frac{\pi }{4} + \frac{{\varphi^{\prime}}}{2}} \right) + 2c^{\prime}{\text{tan}}\left( {\frac{\pi }{4} + \frac{{\varphi^{\prime}}}{2}} \right)$$

4b $$\sigma_{3f}{\prime} = \sigma_{1f}{\prime} \tan^{2} \left( {\frac{\pi }{4} - \frac{{\varphi^{\prime}}}{2}} \right) - 2c^{\prime}\tan \left( {\frac{\pi }{4} - \frac{{\varphi^{\prime}}}{2}} \right)$$

## Analytical solution of matric suction considering transpiration in unsaturated soils

Richards' equation is commonly employed as the governing equation to describe fluid flow in unsaturated soils^35^. Based on this equation, Srivastava and Jim^36^ proposed the closed-form for the one-dimensional form to obtain the matric suction at different depths. Subsequently, Li and Yang^2,4^ used it to solve the matric suction for backfill soil of retaining walls, and carried out an in-depth analysis of the influence parameters. In this study, this idea is referred to obtain matric suction of retaining walls considering transpiration. It is noted that Ng et al.^32^ and Yuan and Lu^37^ have derived the analytical solution of matric suction considering plant transpiration for slope. This analytical solution is modified to suit the matric suction calculation of retaining walls by setting the angle between the soil layer and the horizontal to 0°. The derivation and results are reproduced below.

**Figure 3:**
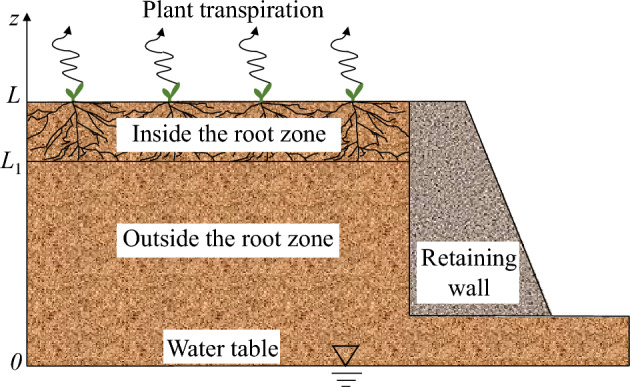
Schematic diagram showing backfill soil.

A semi-infinite homogeneous horizontal soil layer is shown in Fig. 3. It is assumed that the surface of the backfill soil is leveled with the top surface of the retaining wall, and equipotential lines of PWP are parallel to the backfill soil surface, thus it can be treated as a 1D flow perpendicular to the surface.

For a vegetated soil layer, a sink term *S*(*z*) is used to consider root water uptake^38^:

5 $$S\left( z \right) = g\left( z \right)T_{p}$$

where *g*(*z*) is a shape function describing root architecture at a given depth z. *T*_*p*_ is the transpiration rate, which is mainly governed by weather conditions and leaf area. In this study, uniform, triangular, and exponential root architecture are considered. And the water uptake function *S*(*z*) can be rewritten as follows^32,39–41^:

6 $$S\left( z \right) = \left\{ {\begin{array}{ll} {\frac{{T_{p} }}{{L_{2} }}} & {{\text{uniform}}\;{\text{root}}\;{\text{architecture}}} \\ {\frac{{2T_{p} }}{{L_{2} }}\left( {\frac{{z - L_{1} }}{{L_{2} }}} \right)} & {{\kern 1pt} {\text{triangular}}\;{\text{root}}\;{\text{architecture}}} \\ {{\kern 1pt} T_{p} \left[ {\frac{{\exp \left( {z - L_{1} } \right) - 1}}{{\exp \left( {L_{2} } \right) - L_{2} - 1}}} \right]} & {{\text{exponential}}\;{\text{root}}\;{\text{architecture}}} \\ \end{array} } \right.$$

The governing equation can be derived by adding a sink term *S*(*z*) to consider root water uptake^32,39^. It is expressed as

7 $$\frac{\partial }{\partial z}\left( {k\frac{\partial \psi }{{\partial z}}} \right) + \frac{\partial k}{{\partial z}} - S\left( z \right)H\left( {z - L_{1} } \right) = \frac{\partial \theta }{{\partial t}}$$

where *t* is time. *ψ* is the pressure head and is equal to − (*u*_*a*_ − *u*_*w*_)/*γ*_w_ and *γ*_w_ is the unit weight of water. *k* is the water permeability of the soil. *H*(*z* − *L*_1_) is the Heaviside function defined as^42^:

8 $$H\left( {z - L_{1} } \right) = \left\{ {\begin{array}{*{20}c} 1 & {\left( {L_{1} < z \le L} \right)} \\ 0 & {\left( {0 \le z \le L_{1} } \right)} \\ \end{array} } \right.$$

where* L*_*1*_ and* L*_*2*_ are the depth of the outside root zone and root depth, respectively, as shown in Fig. 2.

Equation (7) is a highly nonlinear equation, which can be linearized using Gardner’s^43^ exponential model for the Hydraulic Conductivity Function (HCF) and Soil Water Retention Curve (SWRC). Owing to its simple functional form and reasonable accuracy, Gardner's model is widely used in the literature^1,29^. The HCF and SWRC model can be written as:

9a $$k = k_{s} \exp \left( {\alpha \psi } \right)$$

9b $$\theta = \theta_{r} + \left( {\theta_{s} - \theta_{r} } \right)\exp \left( {\alpha \psi } \right)$$

where *k*_*s*_ is the saturated hydraulic conductivity. *α* is the desaturation coefficient of soil.

Equation (7) can be linearized by implementing Eq. (9):

10 $$\frac{{\partial^{2} k}}{{\partial z^{2} }} + \alpha \frac{\partial k}{{\partial z}} - \alpha S\left( z \right)H\left( {z - L_{1}^{{}} } \right) = \frac{{\alpha \left( {\theta_{s} - \theta_{r} } \right)}}{{k_{s} }}\frac{\partial k}{{\partial t}}$$

The following variable is defined:

11 $$k^{*} = k/k_{s}$$

Substituting Eqs. (11) into (10) yields

12 $$\frac{{\partial^{2} k^{*} }}{{\partial z^{2} }} + \alpha \frac{{\partial k^{*} }}{\partial z} - \frac{{\alpha S\left( z \right)H\left( {z - L_{1}^{{}} } \right)}}{{k_{s} }} = \frac{{\alpha \left( {\theta_{s} - \theta_{r} } \right)}}{{k_{s} }}\frac{{\partial k^{*} }}{\partial t}$$

One dimensional (1D) steady flow is considered, then time is not taken into account, and the corresponding steady-state equation for Eq. (12) is written as

13 $$\frac{{\partial^{2} k^{*} }}{{\partial z^{2} }} + \alpha \frac{{\partial k^{*} }}{\partial z} - \frac{{\alpha S\left( z \right)H\left( {z - L_{1}^{{}} } \right)}}{{k_{s} }} = 0$$

For a given set of boundary conditions, the solution of Eq. (13) can be derived.

Firstly, the upper boundary is given and *q*_*0*_ is the surface flux (evaporation or rainfall flux) under a steady state.

14 $$\left[ {\frac{{\partial k^{*} }}{\partial z} + \alpha k^{*} } \right]_{z = L} = - \alpha q_{0}^{*}$$

where $$q_{0}^{*} = q_{0} /k_{s}$$.

Secondly, bottom boundary is set as that pressure head *ψ* is zero.

15 $$k^{*} \left| {_{z = 0} = \exp \left( {\alpha \psi_{0} } \right)} \right.$$

Using the same procedure as Ng et al.^32^ the solution to Eq. (13) for both the inside and outside root zones with boundary conditions (Eq. (14) and Eq. (15)) can be derived as follows:

Uniform root architecture

16a $$k^{*} = \left\{ {\begin{array}{ll} {A + \frac{{T_{p} }}{{k_{s} }}\left[ {\exp \left( { - \alpha z} \right) - 1} \right]} & {\left( {0 \le z < L_{1} } \right)} \\ {A + \frac{{T_{p} }}{{k_{s} L_{2} }}\left\{ {\left[ {\exp \left( { - \alpha z} \right) - 1} \right]\left( {L - z} \right) + \exp \left( { - \alpha z} \right)\left[ {z - L_{1} - \alpha^{ - 1} \exp \left( {\alpha z} \right){ + }\alpha^{ - 1} \exp \left( {\alpha L_{1}^{{}} } \right)} \right]} \right\}} & {\left( {L_{1} < z \le L} \right)} \\ \end{array} } \right.{\kern 1pt} {\kern 1pt} {\kern 1pt}$$

Triangular root architecture

16b $$k^{*} = \left\{ {\begin{array}{*{20}c} {A + \frac{{2T_{p} }}{{k_{s} L_{2}^{2} }}\left[ {\exp \left( { - \alpha z} \right) - 1} \right]\left[ {\frac{{L^{2} - L_{1}^{2} }}{2} - L_{1} L + L_{1}^{2} } \right]} & {\left( {0 \le z < L_{1} } \right)} \\ {A + \frac{{2T_{p} }}{{k_{s} L_{2}^{2} }}\left\{ \begin{gathered} \left[ {\exp \left( { - \alpha z} \right) - 1} \right]\left( {\frac{{L^{2} - z^{2} }}{2} - L_{1} L + L_{1} z} \right) + \exp \left( { - \alpha z} \right)\left[ {\frac{{\exp \left( {\alpha L_{1} } \right)}}{{\alpha^{2} }}\left( {\alpha L_{1} - 1} \right)} \right. \hfill \\ \left. { - \frac{{\exp \left( {\alpha z} \right)}}{{\alpha^{2} }}\left( {\alpha z - 1} \right) - L_{1} z + L_{1}^{2} - \frac{{L_{1}^{2} - z^{2} }}{2} - \frac{{L_{1} \exp \left( {\alpha L_{1}^{{}} } \right)}}{\alpha } + \frac{{L_{1} \exp \left( {\alpha z} \right)}}{\alpha }} \right] \hfill \\ \end{gathered} \right\}} & {\left( {L_{1} < z \le L} \right)} \\ \end{array} } \right.$$

Exponential root architecture

16c $$k^{*} = \left\{ {\begin{array}{*{20}c} {A + \frac{{T_{p} }}{{k_{s} \exp \left( {L_{2} } \right) - L_{2} - 1}}\left[ {\exp \left( { - \alpha z} \right) - 1} \right]\left\{ {L_{1} - L + \exp \left( { - L_{1} } \right)\left[ {\exp \left( L \right) - \exp \left( {L_{1} } \right)} \right]} \right\}} & {\left( {0 \le z < L_{1} } \right)} \\ {A + \frac{{T_{p} }}{{k_{s} }}\left\{ \begin{gathered} \frac{{\exp \left( { - \alpha z} \right) - 1}}{{\exp \left( {L_{2} } \right) - L_{2} - 1}}\left[ {z - L + \exp \left( { - L_{1} } \right)\left( {\exp \left( L \right) - \exp \left( z \right)} \right)} \right] + \hfill \\ \frac{{\exp \left( { - \alpha z} \right)}}{{\exp \left( {L_{2} } \right) - L_{2} - 1}}\left[ {L_{1} - z - \frac{{\exp \left( {\alpha L_{1} } \right) - \exp \left( {\alpha z} \right)}}{\alpha } - \exp \left( { - L_{1} } \right)\left( {\exp \left( {L_{1} } \right) - \exp \left( z \right)} \right)} \right. \hfill \\ \left. { - \frac{{\exp \left( {z - L_{1} + \alpha z} \right)}}{\alpha + 1} + \frac{{\exp \left( {\alpha L_{1} } \right)}}{\alpha + 1}} \right] \hfill \\ \end{gathered} \right\}} & {\left( {L_{1} < z \le L} \right)} \\ \end{array} } \right.$$

where $$A = \exp \left[ {\alpha \left( {\psi_{0} - z} \right)} \right] + q_{0} \left[ {\exp \left( { - \alpha z} \right) - 1} \right]/k_{s} .$$

Using Eq. (9) and Eq. (16), matric suction (*u*_*a*_-*u*_*w*_) can be derived as:

17 $$\left( {u_{a} - u_{w} } \right) = - \gamma_{w} \alpha^{ - 1} \ln \left\{ {k^{*} } \right\}$$

This equation establishes the relationship between plant transpiration and matric suction, which can be used to solve the spatial distribution of matric suction at a stable state considering plant transpiration.

### Earth pressures considering plant transpiration

In this section, the formulations for matric suction, CMS, and effective stress presented in the previous sections are incorporated into Hooke's law and Rankine's earth pressure theory to determine at-rest, active, and passive earth pressures considering plant transpiration.

#### At-rest earth pressure

Hooke's law is a linear stress–strain constitutive equation, which is commonly used to establish a relationship between vertical and horizontal stress components. In an unsaturated soil layer, Hooke's law can be extended to unsaturated soils by incorporating the effective stress representation i.e., Eq. (1) as follows:

18a $$\varepsilon_{x} = \frac{{\sigma_{x} - u_{a} }}{E} - \frac{\mu }{E}\left( {\sigma_{y} + \sigma_{z} - 2u_{a} } \right) + \frac{{\left( {1 - 2\mu } \right)\chi \left( {u_{a} - u_{w} } \right)}}{E}$$

18b $$\varepsilon_{y} = \frac{{\sigma_{y} - u_{a} }}{E} - \frac{\mu }{E}\left( {\sigma_{x} + \sigma_{z} - 2u_{a} } \right) + \frac{{\left( {1 - 2\mu } \right)\chi \left( {u_{a} - u_{w} } \right)}}{E}$$

18c $$\varepsilon_{z} = \frac{{\sigma_{z} - u_{a} }}{E} - \frac{\mu }{E}\left( {\sigma_{x} + \sigma_{y} - 2u_{a} } \right) + \frac{{\left( {1 - 2\mu } \right)\chi \left( {u_{a} - u_{w} } \right)}}{E}$$

where *ε*_*x*_, *ε*_*y*_ and *ε*_*z*_ are elastic strains in the x, y, and z directions, respectively. σ_x_, σ_y,_ and σ_z_ are elastic total stresses in the x, y, and z directions, respectively. *E* and *μ* represent Young's modulus and Poisson's ratio, respectively. For a homogenous unsaturated layer in a half-space domain, it is reasonable to consider the following simplifying assumptions^1^: The horizontal stresses are equal (*σ*_*x*_ = *σ*_*y*_ = *σ*_*h*_), and the horizontal strains are negligible (*ε*_*x*_ = *ε*_*y*_ = *ε*_*h*_ = 0).

Imposing these assumptions reduce Eq. (18) to:

19 $$\sigma_{h} - u_{a} = \frac{\mu }{1 - \mu }\left( {\sigma_{v} - u_{a} } \right) - \frac{1 - 2\mu }{{1 - \mu }}\chi \left( {u_{a} - u_{w} } \right)$$

where σ_h_ and σ_v,_ are elastic total stresses in the horizontal and vertical directions, respectively.

Substituting Eq. (9) and Eq. (17) into Eq. (2), the CMS become:

20 $$\chi \left( {u_{a} - u_{w} } \right) = - \gamma_{w} \alpha^{ - 1} k^{*} \ln \left\{ {k^{*} } \right\}$$

Substituting Eq. (20) into Eq. (19) yields:

21 $$\sigma_{h} - u_{a} = \frac{\mu }{1 - \mu }\left( {\sigma_{v} - u_{a} } \right) + \frac{1 - 2\mu }{{1 - \mu }}\gamma_{w} \alpha^{ - 1} k^{*} \ln \left( {k^{*} } \right)$$

where $\sigma_{v} - u_{a} { = }\gamma \left( {L - z} \right)$, *γ* is the soil unit weight.

The above equation can be used to determine the at-rest earth pressure distribution along with the depth.

#### Active earth pressure

Active earth pressure is the earth pressure when the backfill is damaged due to the retaining wall moving away from the filling. Rankine’s theory has been widely used in this pressure calculation^1,20^. There are several simplifying assumptions to express this failure in the limit equilibrium analysis. A frictionless boundary is assumed, which allows the lateral movement of a soil mass and reduces the horizontal stress. Further, this assumption enables one to consider the major and minor principal stresses acting along with the vertical and horizontal directions, respectively. According to effective-stress expression Eq. (1), the major and minor principal stresses can be written as:

22a $$\sigma_{3}{\prime} = \left( {\sigma_{h} - u_{a} } \right) + \chi \left( {u_{a} - u_{w} } \right)$$

22b $$\sigma_{1}{\prime} = \left( {\sigma_{v} - u_{a} } \right) + \chi \left( {u_{a} - u_{w} } \right)$$

Implementation of the Mohr–Coulomb failure criterion in the active mode i.e., Eq. (4b) allows defining the stress state at failure as^1^:

23 $$\left( {\sigma_{h} - u_{a} } \right) + \chi \left( {u_{a} - u_{w} } \right) = \left[ {\left( {\sigma_{v} - u_{a} } \right) + \chi \left( {u_{a} - u_{w} } \right)} \right]\tan^{2} \left( {\frac{\pi }{4} - \frac{{\varphi^{\prime}}}{2}} \right) - 2c^{\prime}\tan \left( {\frac{\pi }{4} - \frac{{\varphi^{\prime}}}{2}} \right)$$

Substituting Eq. (20) into Eq. (23) yields:

24 $$\left( {\sigma_{h} - u_{{\text{a}}} } \right){ = }\left( {\sigma_{v} - u_{a} } \right)K_{a} - 2c^{\prime}\sqrt {K_{a} } { + }\left( {1 - K_{a} } \right)\gamma_{w} \alpha^{ - 1} k^{*} \ln \left( {k^{*} } \right)$$

where $K_{a} = \tan^{2} \left( {\frac{\pi }{4} - \frac{{\varphi^{\prime}}}{2}} \right)$, which is called the coefficient of active earth pressure.

#### Passive earth pressure

The passive failure mode is the soil failure of the retaining wall under external loads, such as a soil mass in front of a failing retaining wall or expansive soil mass behind a retaining wall. And the earth pressure acting on the retaining wall is called passive earth pressure. In the passive earth pressure mode, the horizontal pressure is usually greater than the vertical stress induced by soil weight. Considering the Mohr–Coulomb criterion, the state of failure occurs when the horizontal stress reaches a level that the combination of normal and shear stresses exceeds the failure envelope. The fundamental difference between the passive and active mode of failure lies within the direction of principal stresses. The horizontal effective stress is the maximum principal stress and the vertical stress is the minimum principal stress in the passive mode. the major and minor principal stresses can be written as:

25a $$\sigma_{1f}{\prime} = \left( {\sigma_{h} - u_{a} } \right) + \chi \left( {u_{a} - u_{w} } \right)$$

25b $$\sigma_{3f}{\prime} = \left( {\sigma_{v} - u_{a} } \right) + \chi \left( {u_{a} - u_{w} } \right)$$

Substituting Eq. (25) into Eq. (4a) yields:

26 $$\left( {\sigma_{h} - u_{a} } \right) + \chi \left( {u_{a} - u_{w} } \right) = \left[ {\left( {\sigma_{v} - u_{a} } \right) + \chi \left( {u_{a} - u_{w} } \right)} \right]\tan^{2} \left( {\frac{\pi }{4} + \frac{{\varphi^{\prime}}}{2}} \right) + 2c^{\prime}\tan \left( {\frac{\pi }{4} + \frac{{\varphi^{\prime}}}{2}} \right)$$

Substituting Eqs. (20) into (26) yields:

27 $$\left( {\sigma_{h} - u_{a} } \right) = \left( {\sigma_{v} - u_{a} } \right)K_{p} + 2c^{\prime}\sqrt {K_{p} } - \left( {K_{p} - 1} \right)\gamma_{w} \alpha^{ - 1} k^{*} \ln \left( {k^{*} } \right)$$

where $K_{p} = \tan \left( {\frac{\pi }{4} + \frac{{\varphi^{\prime}}}{2}} \right)$, which is called the coefficient of passive earth pressure.

#### The resultant force of earth pressure

**Figure 4:**
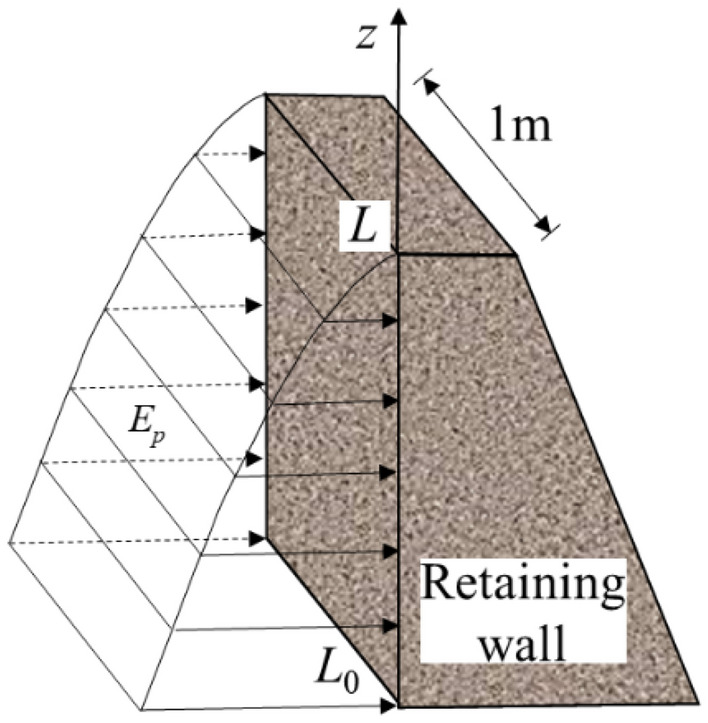
Schematic diagram resultant force calculation.

In the design, the earth pressure will be expressed by the resultant force of earth pressure. The resultant force is the sum of the earth pressures acting on a retaining wall per meter of width, as shown in Fig. 4. In the above study, the distribution of earth pressure considering transpiration has been calculated. Therefore, the resultant force can be expressed by the area enclosed by the earth pressure distribution curve and the vertical coordinate, as shown in Eq. (28).

28 $$E_{p} = \int_{{L_{0} }}^{L} {{\kern 1pt} \left( {\sigma_{h} - u_{a} } \right)_{z} dz}$$

**Figure 5:**
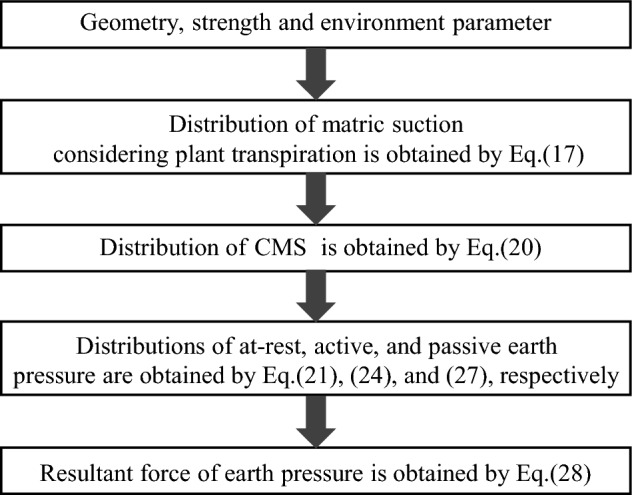
The flowchart of the calculation method proposed by this paper.

Finally, the method framework for efficient calculating earth pressure of retaining wall considering plant transpiration is presented in the Fig. 5. And the earth pressure can be obtained by this method.

### Parametric study

The proposed analytical method framework can be employed to assess the influence of various factors on earth pressures of unsaturated soils subjected to plant transpiration. In this section, we chose three main factors, namely, root architecture, soil type, and transpiration rates, for analysis of its influence on earth pressures. A series of the cases used to analyze these effects are outlined in Table 1.

**Table 1:** Summary of case.

Series	Root architecture type	*T*_*p*_^29,32^ (mm/d)	Soil type	*c*′ (kPa)	$\varphi^{\prime}$(°)	*γ* (KN/m^3^)	*μ*	*k*_*s*_ (m/s)	*α* (m^−1^)
1	Uniform	4.5	Silt^1^	15	30	18	0.35	1e−7	0.20
	Triangular								
	Exponential								
2	Uniform	0	Silt^1^	15	30	18	0.35	1e−7	0.20
		1.5							
		3							
		4.5							
3	Uniform	4.5	Silt^1^	15	30	18	0.35	1e−7	0.20
			Clay^20^	5	25	18	0.20	5e−8	0.13
			Sand^20^	0	35	18	0.25	5e−6	0.70

To simplify the calculation and highlight the effect of transpiration, a hypothetical model is selected and described below. The water table is consistent with the location of the bottom for the retaining wall and is the origin of the ordinate. Moreover, *L* = 5 m is selected as soil thickness. 1 m is chosen as the root zone thickness, which is common for soil with roots^26^. The rate of infiltration *q*_0_ is considered to be constant and equal to zero^1,26^.

#### The influence of root architecture type

Root systems play a vital role in plant life, owing to their function in water absorption from the soil. Root systems have different architectures, which subsequently been associated with variations in water content in the soil under equal conditions^26,32,44^. Therefore, As the most common root architectures, the Uniform, Triangular, and Exponential root architectures are considered to study the effect of the root architecture types on the earth pressure, and the parameters are shown in series 1 in Table 1.

**Figure 6:**
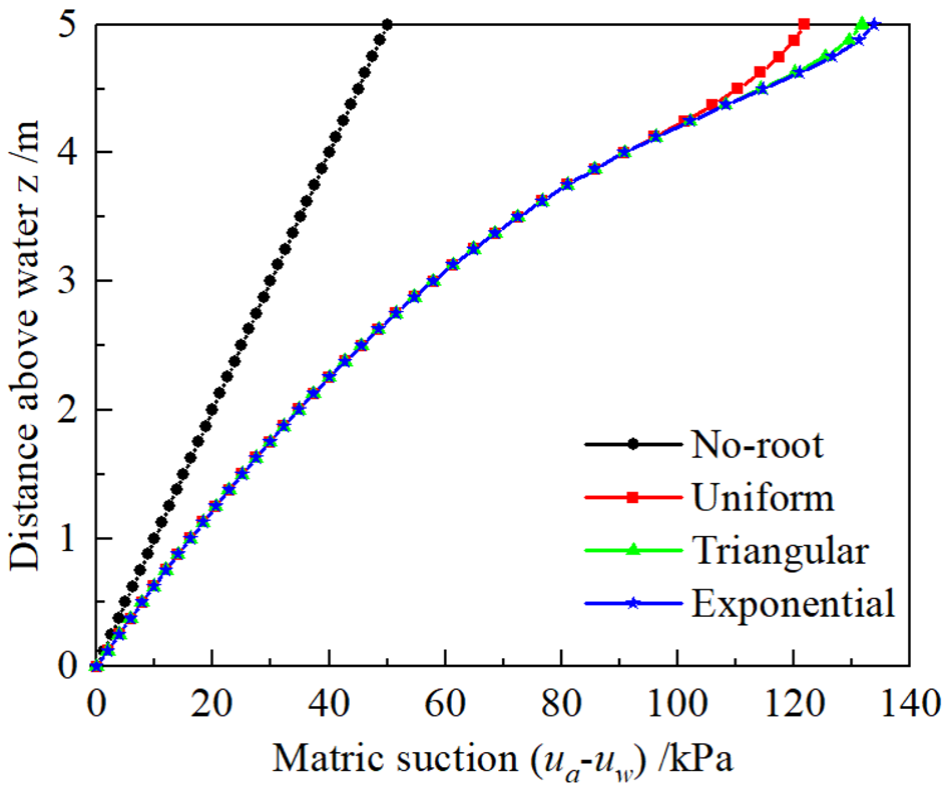
Profiles of matric suction distributions for root architecture types.

To investigate the mechanism underlying plant effect on earth pressures, it is necessary to analyze the matric suction and CMS, as these represent the bridge through which plants influence the earth pressures. Variations matric suction with z for different root architecture types are depicted in Fig. 6. Summarily, it shows that the matric suction increases after considering plant transpiration. The maximum matric suction is about 1.7 times larger than that in the no-plant soil layer at the soil surface. Notably, different matric suctions are evident for different root architecture types, which is because the different types means the different water absorption capacities along the depth (Eq. (7)) and was also confirmed by relevant physical model tests such as centrifuge tests^45^. The matric suction of the exponential root is the maximum, and it is close to that of the triangular root. Conversely, a uniform root has a smaller matric suction than another root. However, it is no difference in matric suction distributions among three root architectures beyond root depth.

**Figure 7:**
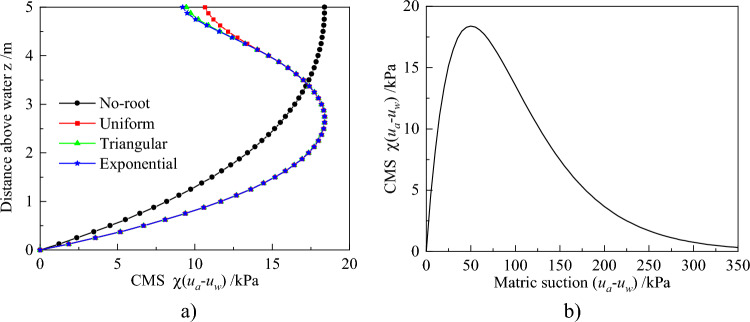
Profiles of CMS distribution for root architecture types: (**a**) For different root architecture types along with z. (**b**) For silt along with matric suction.

Variation of the matric suction distribution changes CMS. Figure 7a depicts the variation of CMS with z for different root architecture types, and its variation with matric suction for silt is depicted in Fig. 7b. Notably, CMS distributions are significantly different between plant transpiration and lack of it (Fig. 7a). All of CMS is only equal at z = 3.3 m, but when z < 3.3 m, the CMS taken into account plant transpiration is large and reversed in other areas. Meanwhile, the CMS distribution, considering no plant transpiration, constantly increases always along with z. However, the other CMS distributions decrease in the upper soils when z is greater than 2.63 m. This is attributed to the fact that CMS first reaches the peak point, then gradually decreases with increasing matric suction (Fig. 7b). When z = 2.63 m, the matric suction considering plant transpiration increases up to 50 kPa, which corresponds to the peak of CMS. Further, CMS for different root architecture are the same in the no-root zone. Triangular and exponential root architectures generate almost the similar distribution in the root zone, and these are lower than those observed in the uniform root architectures.

**Figure 8:**
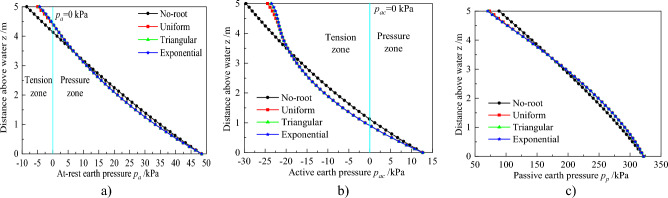
Profiles of Earth pressure distributions for root architecture types: (**a**) At-rest earth pressure. (**b**) Active earth pressure. (**c**) Passive earth pressure.

The above results revealed that different root architectures affect matric suction and CMS of soil. From this, the evolution law of earth pressure can be deeply analyzed under different root architectures. Figure 8 shows the at-rest, active, and passive earth pressures for the different root architectures.

Figure 8a displays that there are two-zone i.e. tension and pressure, which is because the action of matric suction (Eq. 21). The at-rest earth pressure does not consider the stress in the tension zone, because it is generally deemed that the soil cannot bear the tension^1,4,20^. However, it is also worthy of attention due to the fact that it is highly likely to develop a crack under the tension stress. The tension zone becomes smaller, considering plant transpiration, while the location of *P*_*a*_ = 0 kPa increases to about z = 4.4 m, indicative of a smaller potential area for soil cracking. In the pressure zone, there is a significant difference in at-rest earth pressure between considering plant transpiration and without it. When z > 3.3 m, the pressure increases after considering plant transpiration, while an opposite trend is recorded in the other zones. Notably, there are no significant differences in the distribution across different root architectures, while the earth pressure is equal in the no-root zone. This phenomenon can be explained by the fact that they are similar to the variation of CMS with z (Fig. 7a). Further, we used Eq. (30) to calculate the resultant forces of at-rest earth pressure, and the results are 94.35, 90.90, 90.93, and 90.93 kN for no plant transpiration, uniform root, triangular root, and exponential root, respectively. Based on these findings, it is evident that the resultant forces decrease after considering plant transpiration, with small differences among the three root architectures.

Profiles of distribution of the active earth pressures are displayed in Fig. 8b. There are also tension and pressure zone. The locations of *P*_*ac*_ = 0 kPa are 0.9 m and 1.1 m when considering and not considering plant transpiration, respectively. Notably, the locations of *P*_*ac*_ = 0 kPa are equal for the three root architectures, because the matric suction is the same in this zone (Fig. 7a). This also explains why active earth pressure distributions and the resultant forces are the same. The resultant forces, when considering and not considering plant transpiration are 6.83 and 5.47kN, respectively, which implies that the resultant forces decrease after considering plant transpiration.

The distribution of the passive pressures is summarized in Fig. 8c. Notably, this pressure is different from other earth pressure in that there is not a tension zone. This is confirmed by the essence of passive pressures that passive pressure is a kind of pressure when retaining walls are subjected to an external load, such as a soil mass in front of a failing retaining wall or expansive soil mass behind a retaining wall. The pressures decrease when z > 3.3 m, but increase in another zone when considering plant transpiration. This indicates that plant transpiration reduces and increases earth pressure in the upper and lower parts, of the retaining wall, respectively. The resultant forces are 1066.90, 1072.09, 1070.97, and 1070.79kN for no plant transpiration, uniform root, triangular root, and exponential root, respectively. This means that soil with plants can bear greater force when the retaining wall exhibits passive failure mode. And it also indicates that uniform root has a greater and significant influence than other root architectures, with that of other root architectures found to be approximate.

#### The influence of transpiration rate

Transpiration rate represents the amount of water that is extracted by roots per unit time, which means different rate corresponds to different change in the water content and mechanical properties of soil. In this section, we adopted *T*_*p*_ = 0, 1.5, 3.0, and 4.5 mm/d, to analyze the influence of transpiration rates on earth pressure as shown in series 2 in Table 1. *T*_*p*_ = 0 mm/d means that the soil is not influenced by plants and the results of matric suction are the same with no plant.

**Figure 9:**
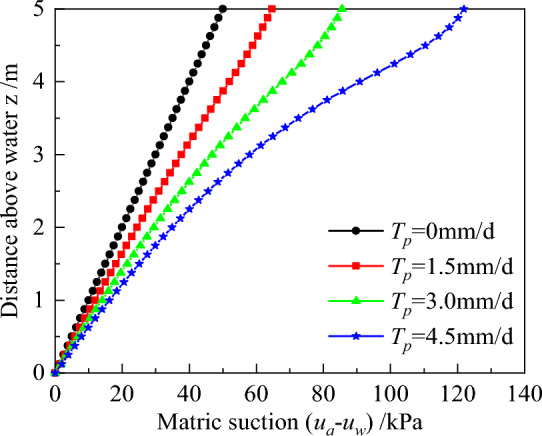
Distributions of matric suction for different transpiration rate.

Variation of matric suction with z for different transpiration rates are depicted in Fig. 9. From the results, it is clear that the matric suction increases with increases transpiration rate. The increment of matric suction is 14.67, 20.83, and 36.36 kPa at the soil surface, respectively, namely rise with increasing the rates. This is explained by the fact that an increase in transpiration rate causes a corresponding increase in water absorption capacity in plants thereby accelerating water loss from the soil^46^. Results further show that the growth of matric suction is greatest near the junction of the no-root zone and the root zone, especially when *T*_*p*_ = 4.5 mm/d. This is attributed to the fact that a higher matric suction, makes it the more difficult for plants to absorb water from the soil.

**Figure 10:**
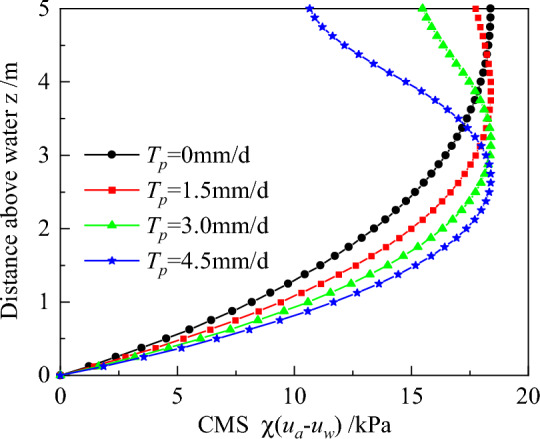
Distributions of CMS for different transpiration rate.

Variations in CMS with z for different transpiration rates is depicted in Fig. 10. Summarily, CMS increases and decreases in the lower and upper soil layers, respectively, after considering plant transpiration. The locations of the difference in this law are z = 4.3, 3.8, and 3.4 m for *T*_*p*_ = 1.5, 3.0, and 4.5 mm/d, respectively, indicating that the locations decline with transpiration rates. This phenomenon can be explained by the fact that higher transpiration rates, influence on matric suction, and the location is lower when the matric suction is up to 50 kPa that is the matric suction when CMS is up to maximum. These are summarized in Fig. 9 and Fig. 8b.

**Figure 11:**
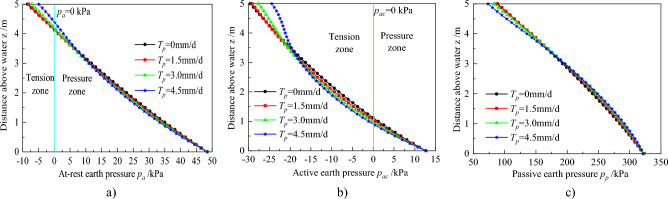
Profiles of earth pressure distributions for different transpiration rate: (**a**) At-rest earth pressure, (**b**) Active earth pressure, (**c**) Passive earth pressure.

Profiles of earth pressure distributions are illustrated in Fig. 11. The tension and pressure zones are depicted in Fig. 11a. The partition locations are 4.14, 4.13, 4.19, 4.40 m for *T*_*p*_ = 0, 1.5, 3.0 and 4.5 mm/d, respectively, indicating that the tension zone has a small increase when *T*_*p*_ increases to 1.5 mm/d, although this zone decreases when *T*_*p*_ increases to 3.0 and 4.5 mm/d. In addition, it is also evident that the pressure earth decreases in the pressure zone when *T*_*p*_ increases to 1.5 mm/d. Nonetheless, the earth pressure first decreases, then increases for others *T*_*p*_. The locations where earth pressure is equal to no plant are z = 4.36, 3.83, and 3.42 m for* T*_*p*_ = 1.5, 3.0, and 4.5 mm/d, respectively. In addition, the resultant forces which are 94.35, 92.23, 90.95 and 90.90 kN for* T*_*p*_ = 0, 1.5, 3.0 and 4.5 mm/d, respectively. Notably, these forces decrease with an increase in *T*_*p*_ but are similar when *T*_*p*_ = 3.0 and 4.5 mm/d.

A summary of active earth pressure distributions is illustrated in Fig. 11b. There are also tension and pressure zones, while the locations of *P*_*ac*_ = 0 kPa are 1.12, 1.04, 0.97 and 0.91 m for *T*_*p*_ = 0, 1.5, 3.0 and 4.5 mm/d, respectively. In tension zone, the maximum of pressure appears at the surface, and decreases with an increase in *T*_*p*_. From this results, it is implied that the cracks are deeper, but the soil near the surface may crack more narrowly with an increase in *T*_*p*_, when occurs active failure mode. In pressure zone, the earth pressure decreases with an increase in *T*_*p*_ anywhere. Meanwhile, the resultant forces for different *T*_*p*_ are 6.83, 6.31, 5.86, and 5.47 kN. Based on these results, it is evident that the pressures decline with increasing *T*_*p*_, mainly because the matric suctions are less than 50 kPa where CMS increase with increasing matric suction (Fig. 7b).

Profiles of passive pressure distribution are illustrated in Fig. 11c. Summarily, the pressures first increase, then decrease with z, after increasing *T*_*p*_. The locations of the difference in the law are z = 4.36, 3.83, and 3.42 for *T*_*p*_ = 1.5, 3.0, and 4.5 mm/d, respectively, indicating that a larger transpiration rate is associated with a smaller increase zone. The resultant forces are 1066.90, 1075.66, 1078.15, and 1072.09 kN for *T*_*p*_ from 0 to 4.5 mm/d, which means that the resultant forces first increase, then decrease with increasing *T*_*p*_. However, it is found that the resultant forces are larger than no plant transpiration. From these analysis, it is concluded that the stability of retaining wall is more favorable under passive failure model when the transpiration rate is 3.0 mm/d.

#### The influence of soil type

The retaining wall exhibit various types of backfill, while soils have different mechanical properties due to their variations in their internal structures and components, indicating that the soil shows different hydraulic characteristics under the influence of plant. In this section, the three hypothetical soil types, namely Clay, Silt, and Sand, are considered from existing literature^1,20^ corresponding to the parameters of series 3 shown in Table 1.

**Figure 12:**
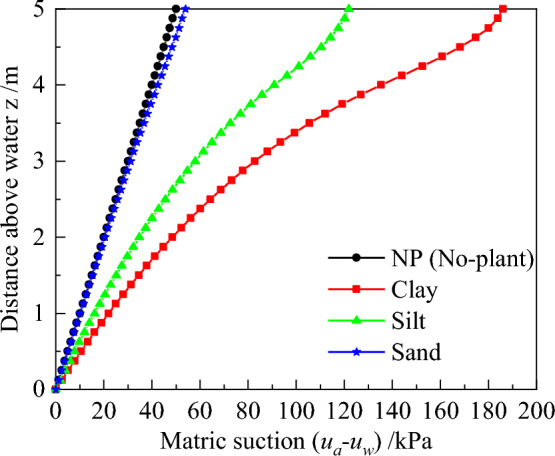
Profiles of matric suction distribution for different soil type.

Variation in matric suction with z for different soil types are illustrated in Fig. 12. Summarily, there is a small change in matric suction of sand and a significant difference for silt and clay after considering plant transpiration. The maximum of matric suction for sand, silt, and clay is 53.9, 121.8, and 186.0 kPa, respectively. This is attributed to the fact that sand has a larger particle size than other soils and changes water content have little effect on the inter-granular force.

**Figure 13:**
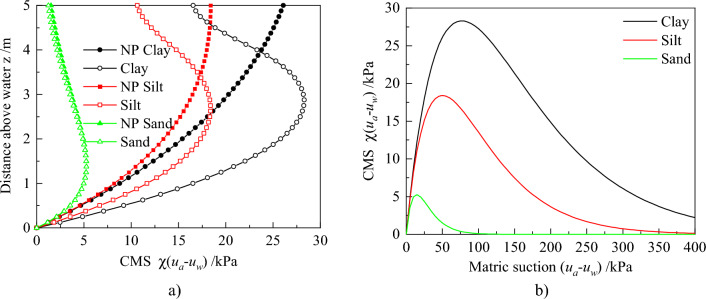
Profiles of CMS distributions for different soil type: (**a**) CMS with z, (**b**) CMS with matric suction.

Figure 13a depicts the variation of CMS with z for different soils under two conditions with and without plant transpiration. Notably, there are significant differences in CMS distribution for different soils. The order of CMS from small to large is sand, silt, and clay, which can be explained by Fig. 13b. Moreover, the maximum CMS for clay, silt, and clay, are 28.3, 18.4, and 5.3 kPa, respectively, corresponding to matric suction of 77.0, 50.0, and 14.5 kPa. The CMS of sand reaches the inflection point faster with the change of matric suction. When it is the same soil under different plant transpiration, the CMS of clay has the most significant difference and the sand has the smallest difference. That may also be explained by previous reasons.

**Figure 14:**
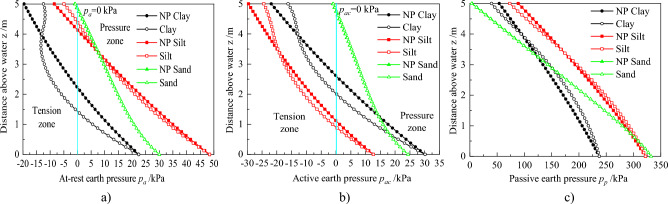
Profiles of earth pressure distributions for different soil type: (**a**) At-rest earth pressure, (**b**) Active earth pressure, (**c**) Passive earth pressure.

The at-rest, active, and passive earth pressures for the different soil under two conditions with or without plant transpiration are depicted in Fig. 14. Presence of tension and pressure zones is confirmed by Fig. 14a. Notably, clay and sand have the largest and smallest tension zones, respectively. For clay, the locations of *p*_*c*_ = 0 kPa are 1.43 and 2.22 m when considering and not considering plant transpiration, respectively. Resultant forces are 14.76 and 23.58 kN when considering and not considering plant transpiration, respectively. These indicates that the earth pressures decreases after considering plant transpiration. The earth pressures for silt have been analyzed in the “The influence of root architecture type”. For the sand, we found little differences with the resultant forces of 63.70 and 63.35 kN when considering and not considering plant transpiration, respectively. Notably, the difference was only 0.35 kPa.

Figure 14b shows the distribution of the active earth pressures. It is clear that the locations order of *p*_*c*_ = 0 kPa is silt, clay, and sand. Silt and sand have the largest and smallest tension zone, respectively. There exist different laws for different soils. For sand, the pressure first decreases and increases after considering plant transpiration, while the other soils only exhibit the law of decrease. The resultant forces are 27.50 and 37.64 kN for clay, 5.47 and 6.83 kN for silt, 48.64 and 48.27 kN for sand, under considering plant transpiration or not, respectively. The resultant forces in clay and silt decrease by 10.14 and 1.36 kN, while sand increases by 0.37 kN. This means that plant transpiration has the significantly influences active earth pressure of in clay.

Profiles of passive earth pressure are summarized in Fig. 14c. The maximums of passive earth pressure are 237.45, 321.96, and 332.12 kPa for clay, silt, and sand, respectively, indicating that the maximum of sand is the largest and the clay is the smallest. However, it is also clear that the minimum of sand is the smallest and the silt is the largest. The law is revealed that the passive earth pressure has the fastest reduction speed with increasing z for sand. Moreover, pressure first increases, then decreases with z after considering plant transpiration, while the locations of change are 3.96, 3.42, and 1.41 m for clay, silt, and sand. The resultant forces for clay are 784.95 and 752.93 kN, when considering and not considering plant transpiration, respectively, whereas those for silt are 1072.09 and 1066.90 kN. However, the resultant forces for sand are 876.15 and 877.69 kN when with and without plant transpiration, respectively, and the difference is just 1.54 kN. This is because the location of the law change is 1.41 m and the reduction zone is larger than the increased zone after considering plant transpiration.

## Conclusions

Analysis of earth pressures in retaining wall usually consider many factors that change the moisture content of the backfill, e.g., rainfall, evaporation, groundwater, and so on. Notably, plant transpiration is rarely considered. This paper addressed this problem by proposing a calculation method, then carrying out the parameters study. The main conclusions are as follows:

1. This paper presents an analytical method framework to determine earth pressures of unsaturated soils considering plant. Specifically, a closed-form solution of matric suction considering plant proposed by Ng et al.^32^ is limited to adapt the calculation of matric suction for the backfill soil. Next, the solution is used to extend the traditional earth pressure theory, and the relationship between plant transpiration and earth pressure is built and the method is given that can analyze the influence of plant transpiration on at-rest, active and passive earth pressures.
2. At-rest earth pressure is close for uniform, triangular and exponential root architecture types, whereas active earth pressure is the same for different root architecture types. Notably, passive earth pressure is different from other earth pressure, as evidenced by lack of a tension zone. The pressures first increase, then decrease with z, while the resultant forces are close to triangular and exponential root architecture types.
3. The tension zone for at-rest earth pressure first increases, then decreases with increase in transpiration rates. Conversely, the resultant forces not only decrease with increasing *T*_*p*_ but are also similar when *T*_*p*_ = 3.0 and 4.5 mm/d. Active earth pressure and its resultant forces decrease with increasing transpiration rates. On the other hand, passive pressures first increase, then decrease with z after increasing *T*_*p*_, while the resultant forces are larger than when plant transpiration is not considered.
4. Clay has the largest tension zone under at-rest earth pressure, while sand has the smallest. There is a small difference in the pressure of sand, and its resultant force exhibits a little increased after considering plant transpiration. However, the resultant forces in other soils exhibit a marked decrease. Active pressure for sand first decreases and increases after considering plant transpiration, while other soils is only a decrease. Meanwhile, the resultant force for sand increases and is opposite to that of the other two soils. Passive pressure for different soils first increase, then decreases with z after considering plant transpiration. Sand has the fastest reduction in passive earth pressure with increasing z. In addition, clay and silt exhibit a marked decrease in resultant forces, while sand only exhibits a small increase.
5. There are some interesting ideas which can be explored in the future work based on this study. To comprehensively real the influence of the plant transpiration on the earth pressure, the change of transpiration rates with time and the difference of plant spacing can be considered to reveal long-term evolution of earth pressure over time. Meanwhile, the influence of plant transpiration on earth pressure can also be investigated by different methods such as experiment and numerical simulation.

## Date availability

The data used to support the findings of this study are included in the article.
